# Prevalence of visual impairment, cataract surgery and awareness of cataract and glaucoma in Bhaktapur district of Nepal: The Bhaktapur Glaucoma Study

**DOI:** 10.1186/1471-2415-11-2

**Published:** 2011-01-21

**Authors:** Suman S Thapa, Rosa VD Berg, Shankar Khanal, Indira Paudyal, Pooja Pandey, Nhukesh Maharjan, Shankha N Twyana, Govinda Paudyal, Reeta Gurung, Sanduk Ruit, Ger HMBV Rens

**Affiliations:** 1 Nepal Glaucoma Eye Clinic, Tilganga Institute of Ophthalmology, Kathmandu, Nepal; 2 Vrije Universiteit Amsterdam, Amsterdam, The Netherlands; 3 Central Department of Statistics, Tribhuvan University, Kathmandu, Nepal; 4 Helen Keller International, Kathmandu, Nepal; 5 Tilganga Institute of Ophthalmology, Kathmandu, Nepal

## Abstract

**Background:**

Cataract and glaucoma are the major causes of blindness in Nepal. Bhaktapur is one of the three districts of Kathmandu valley which represents a metropolitan city with a predominantly agrarian rural periphery. This study was undertaken to determine the prevalence of visual impairment, cataract surgery and awareness of cataract and glaucoma among subjects residing in this district of Nepal.

**Methods:**

Subjects aged 40 years and above was selected using a cluster sampling methodology and a door to door enumeration was conducted for a population based cross sectional study. During the community field work, 11499 subjects underwent a structured interview regarding awareness (heard of) and knowledge (understanding of the disease) of cataract and glaucoma. At the base hospital 4003 out of 4800 (83.39%) subjects underwent a detailed ocular examination including log MAR visual acuity, refraction, applanation tonometry, cataract grading (LOCSΙΙ), retinal examination and SITA standard perimetry when indicated.

**Results:**

The age-sex adjusted prevalence of blindness (best corrected <3/60) and low vision (best corrected <6/18 ≥3/60) was 0.43% (95%C.I. 0.25 - 0.68) and 3.97% (95% C.I. 3.40 - 4.60) respectively. Cataract (53.3%) was the principal cause of blindness. The leading causes of low vision were cataract (60.8%) followed by refractive error (12%). The cataract surgical coverage was 90.36% and was higher in the younger age group, females and illiterate subjects. Pseudophakia was seen in 94%. Awareness of cataract (6.7%) and glaucoma (2.4%) was very low. Among subjects who were aware, 70.4% had knowledge of cataract and 45.5% of glaucoma. Cataract was commonly known to be a 'pearl like dot' white opacity in the eye while glaucoma was known to cause blindness. Awareness remained unchanged in different age groups for cataract while for glaucoma there was an increase in awareness with age. Women were significantly less aware (odds ratio (OR): 0.63; 95%, confidence interval (CI): 0.54 - 0.74) for cataract and (OR: 0.64; 95% CI: 0.50 - 0.81) for glaucoma. Literacy was also correlated with awareness.

**Conclusion:**

The low prevalence of visual impairment and the high cataract surgical coverage suggests that cataract intervention programs have been successful in Bhaktapur. Awareness and knowledge of cataract and glaucoma was very poor among this population. Eye care programs needs to be directed towards preventing visual impairment from refractive errors, screening for incurable chronic eye diseases and promoting health education in order to raise awareness on cataract and glaucoma among this population.

## Background

Cataract is the major cause of blindness worldwide. It is estimated that 41.8% of all global blindness is caused by cataract [1]. Glaucoma is the second leading cause of visual loss in the world. Quigley estimates that there will be 60.5 million people with glaucoma in 2010 and Asians are expected to represent 47% of those with glaucoma [2]. World Health Organization has estimated the prevalence of blindness amongst people over fifty years in South East Asia as 3.4% [3]. Nepal Blindness Survey conducted in 1981 reported the overall prevalence of blindness in Nepal as 0.84% and in subjects more than 45 years of age as 3.8% [4]. After this survey few population based studies have been undertaken in Nepal [5-7].

Implementing health care programs in a given community and promoting awareness of common eye diseases can bring forth people to have an eye examination. This could result in the early diagnosis, treatment and reduction of visual impairment and blindness from eye diseases. Studies undertaken in the region have revealed a poor awareness of eye diseases among the general population [8-10]. It has also been reported that approximately 50% of patients with glaucoma were unaware of their condition at the time of diagnosis [11] and present in the advanced stage of the disease [12,13].

The aim of this paper is to describe the prevalence and causes of visual impairment, to calculate the cataract surgical coverage and to determine the awareness and knowledge of cataract and glaucoma among the population aged 40 years and above. This is the first study to determine the reduction of visual impairment in one of the districts within Kathmandu valley. It is also the first study to determine the awareness of cataract and glaucoma in a Nepali population.

## Methods

The study was designed as a population based cross-sectional study. A sample size of 4758 was calculated after assuming a 3% prevalence of blindness, a relative precision of 25%, compliance of 85% and a design effect of 2. An assumption of 3% was based on previous studies conducted in Nepal. According to the 2001 Census, the population of Bhaktapur was 225,461 with a population density of 1,895 per square kilometer [14]. The sampling frame comprised of 161 wards, with an estimated total population of 48,223 people above the age of 40 years residing in this area. The survey involved selection of 4800 subjects, 40 years and above, using WHO 30 cluster sampling procedure [15].

At the first stage, a list of all wards or clusters from 16 Village Development Committees (VDC) and 2 municipalities were obtained from National Census data [14]. Thirty clusters were randomly selected and subjects sampled with probability proportionate to size. While undertaking the census, community field workers interviewed 11,499 subjects enlisted during the field work. Six community field workers were trained in the interview procedures by the principal investigator and conducted a structured-interview regarding awareness for cataract and glaucoma. The questionnaire was first designed in English and translated into Nepali, the national language of Nepal. Subjects were asked if they had heard of cataract and glaucoma. Those who responded with a 'yes' were termed as being 'aware' and were further encouraged to explain what they knew about those conditions. Subjects with responses which matched the list of answers in the questionnaire were regarded to have 'knowledge' of the eye disease. Demographic details as well as all responses were documented. A pilot study was conducted on volunteers and minor modifications were made later in order to finalize the interview questionnaire.

At the second stage, a database was prepared where names of eligible subjects were recorded. 4800 subjects were selected using EPI-INFO software, version 3.5.1 (Centers for Disease Control and Prevention, Atlanta, GA). The selected subjects were then revisited by the community field staff and referred to Tilganga Institute of Ophthalmology (TIO) for a comprehensive eye examination.

The distance and near visual acuity (VA) both presenting and best corrected after refraction were measured using logarithm of minimum angle of resolution (log MAR) tumbling E charts (Precision Vision, USA) placed at 4 meters. Objective refraction was done using a streak retinoscope (Beta 200 Heine, Germany) followed by a subjective refraction. The log MAR chart was moved to 1 meter if the subject was unable to read the top line, and acuity was tested again. If VA could not be measured then counting fingers at 1 meter, hand movements and light perception were sequentially checked. A detailed ocular examination with slit-lamp biomicroscope (Haag Streit BQ 900) was carried out. This included measurement of intraocular pressure with Goldmann applanation tonometer and gonioscopy with 4 mirror Zeiss gonioscope. The angle was graded according to the Shafer system [16]. Lens was examined after pupilary dilatation (unless contra-indicated) and cataract graded using Lens Opacities Classification System (LOCS) II. Stereoscopic fundus examination was done using 90-diopter lens and indirect ophthalmoscopy using 20-diopter lens. Automated visual field test using the SITA Standard 24-2 program (Model 750, Humphrey Instruments, San Leandro, CA, USA) was performed for all the subjects who were glaucoma subjects and with diseases such as glaucoma, optic atrophy, and retinitis pigmentosa.

Visual impairment (VI), blindness and low vision were defined as per International Classification of Diseases 10^th ^edition (ICD -10) [17]. The International Classification of Diseases 10^th ^edition (ICD -10) defines visual impairment as VA of less than 6/18 (20/60, 0.3) in the better eye with the best correction [17]. Visual impairment has been categorized to blindness and low vision. A VA of less than 3/60 (20/400, 0.05) with best correction or a visual field less than 10° from fixation in the better eye has been considered blindness. Low vision has been defined as a best corrected VA of less than 6/18 (20/60, 0.3), but not less than 3/60 (20/400, 0.05) in the better eye. Presenting VA has also been used to describe visual impairment within the study sample.

Diagnosis was recorded using International Classification of Diseases-ninth revision (ICD-9). If more than one disease was present, the disease most likely to have a significant effect on vision was considered as the cause for blindness.

Cataract blindness burden was defined as a sum of those people already operated for cataract in both eyes and the unoperated cataract blind. It was not possible to obtain the preoperative vision of an already operated eye and assumption was made that both eyes were blind preoperatively if both eyes were operated for cataract, or if one eye was operated and the other eye was blind at the time of our examination. Cataract surgical coverage was calculated as number of bilaterally blind cataract cases operated divided by the number who could have been operated. The denominator includes the already operated bilateral blind (the numerator) plus the unoperated bilaterally blind with cataract being the principal cause of blindness in at least one eye.

Literacy was determined by asking subjects whether they could read and write. Those who could were considered to be literate. This study was approved by the Institutional Review Board and Ethics Committee of TIO and conducted in accordance with declaration of Helsinki. Informed consent was obtained from all subjects after being explained in detail about all the procedures to be undertaken. The informed consent was written in the vernacular. The consent form was read out for those unable to read. Upon agreement by the subject, they were asked to sign the consent form. For those unable to sign, thumb impressions were taken.

Descriptive statistical measures were presented to summarize data. Univariate and multivariable logistic regression analysis were applied. Odds ratio (OR) were computed. Statistical analysis was carried out using STATA software version 9.0.

## Results

### Participants

Out of 4800 enumerated subjects, 4003 were examined (response rate of 83.39%). Data was incomplete for 24 subjects, leaving 3979 subjects for analysis (82.90%). Mean age of study population was 55.10 years (SD 11.50), more females were examined (54.49%) and 2119 subjects (53.25%) were illiterate. More illiterate subjects refused to participate in the study. The demographics of examined subjects are presented in Table 1.

**Table 1:** Demographic comparison of enumerated and examined subject

	Enumerated		Examined		
**Age group**	**No.**	**%**	**No.**	**%**	**p**

40-49	1849	38.7	1481	37.22	

50-59	1245	25.9	1089	27.37	

60-69	951	18.8	835	20.99	

70-79	591	12.3	477	11.99	

>80	205	4.3	97	2.44	< 0.001

**Sex**					

Male	2314	48.2	1811	45.51	

Female	2486	51.8	2168	54.49	0.012

**Education**					

Literate	2131	44.4	1860	46.75	

Illiterate	2688	55.6	2119	53.25	0.018

**Total**	4800	100.0	3979	100.00	

### Prevalence and causes of visual impairment

Overall prevalence of VI at presentation was 18.57% (95% C.I. 17.37 - 19.82) and after best correction 4.4% (95% CI: 3.78 - 5.82). VA measurements are presented in Table 2. There were 710 (17.83%) subjects with low vision at presentation. Vision improved to better than 6/18 in 552 (77.75%) in subjects after refraction, remaining 158 (3.97%) had low vision after correction. Prevalence of blindness at presentation was 0.73% and after correction 0.43%. 17 were blind even after best correction and there were 10 males (58.82%), 7 females (41.18%) with a mean age of 71.29 (SD 11.21) years. Out of 17, 9 (52.94%) were literate.

**Table 2:** Presenting visual acuity and best corrected visual acuity (better eye)

	Presenting visual acuity (better eye)				Best corrected visual acuity (better eye)			
	**> 6/18**	**6/18 - 3/60**	≤ **3/60**	**p**	**> 6/18**	**6/18 - 3/60**	≤ **3/60**	

**Sex**								
Male	1502 (82.94%)	295 (16.29%)	14 (0.77%)		1737 (95.91%)	64 (3.53%)	10 (0.55%)	
Female	1738 (80.17%)	415 (19.14%)	15 (0.69%)	0.063	2067 (95.34%)	94 (4.34%)	7 (0.32%)	0.241

**Age (Years)**								
40-49	1381(93.25%)	97(6.55%)	3(0.20%)		1471 (99.32%)	9 (0.61%)	1 (0.07%)	
50-59	949(87.14%)	139(12.76%)	1(0.09%)		1075 (98.71%)	13 (1.19%)	1 (0.09%)	
60-69	619(74.13%)	207(24.79%)	9(1.08%)		792 (94.85%)	39 (4.67%)	4 (0.488%)	
70-79	257(53.88%)	207(43.40%)	13(2.73%)		400 (83.86%)	68 (14.26%)	9 (1.89%)	
≥ 80	34(35.05%)	60(61.86%)	3(3.09%)	<0.001	66 (68.04%)	29 (29.90%)	2 (2.06%)	<0.001

**Education**								
Literate	1516(81.51%)	328(17.63%)	16(0.86%)		1781 (95.75%)	70 (3.76%)	9 (0.48%)	
Illiterate	1724(81.36%)	382(18.03%)	13(0.61%)	0.063	2023 (95.47%)	88 (4.15%)	8 (0.38%)	0.723

Prevalence of blindness (presenting visual acuity, better eye): 0.73% (95% C.I. 0.49 - 1.04)

Prevalence of low vision (presenting visual acuity, better eye): 17. 83% (95% C.I. 16.65 - 19.05)

Prevalence of blindness (best corrected visual acuity, better eye): 0.43% (95%C.I. 0.25 - 0.68)

Prevalence of low vision (best corrected visual acuity, better eye): 3.97% (95% C.I. 3.38 - 4.62)

Causes of VI are presented in Table 3. Cataract was the leading cause of blindness in all four groups. Leading causes of low vision were cataract (60.8%) and refractive error (12%). In subjects with bilateral blindness cataract was responsible in 16 (47.1%) eyes while corneal scars and retinal disorders accounted for 5 (14.7%) eyes each. 15 subjects were blind from the same cause in both eyes, cataract was responsible in 8 (53.3%) subjects while corneal scar and retinal disorders were accountable in 2 (13.3%) subjects each.

**Table 3:** Causes of visual impairment (best corrected, better eye)

Cause	Low vision	Unilateral blindness	Bilateral blindness
Cataract	96 (60.8%)	53 (37.1%)	16 (47.1%)

Retinal disorder	18 (11.4%)	21 (14.7%)	5 (14.7%)

Corneal scar	4 (2.5%)	18 (12.6%)	5 (14.7%)

Refractive error	19 (12.0%)	10 (7.0%)	3 (8.8%)

Phthisis bulbi	0 (0.0%)	14 (9.8%)	2 (5.9%)

Trauma	0 (0.0%)	11 (7.7%)	1 (2.9%)

Glaucoma	4 (2.5%)	5 (3.5%)	2 (5.9%)

Surgical complication	5 (3.1%)	4 (2.8%)	0 (0%)

Amblyopia	1 (0.6%)	3 (2.1%)	0 (0%)

Optic atrophy	1 (0.6%)	3 (2.1%)	0 (0%)

Undetermined	2 (1.3%)	1 (0,7)	0 (0%)

PCO	6 (3.8%)	0 (0%)	0 (0%)

Aphakia	2 (1.3%)	0 (0%)	0 (0%)

Total	158 (100%)	143 (100%)	34 (100%)

PCO Posterior Capsule Opacification

Since the number of blind people was very few (17 out of 3979) we combined two groups (low vision and blindness) together for analysis. The results for VI are presented in Table 4. VI had a positive association with increasing age (p < 0.001) in each age group. Odds ratio (OR) increased from 2.06 (1.57 - 2.69) for 50 - 59 years age group to 25.94 (16.30 - 41.30) for subjects aged ≥80 years. The odds of VI at presentation was significantly higher in females (OR: 1.30, 95% C.I.: 1.09 - 1.55) but after best correction there was no difference. Literacy had no direct association with VI.

**Table 4:** Effect of age, sex and literacy on visual impairment after best correction

Variable	Normal (%)	Visual **impairment **^**φ **^ (%)	Univariate		Multivariable	
			
			OR(95% CI)	P value	OR(95% CI)	P value
**Age(years)**						
40 - 49	1471(38.67)	10(5.71)	1.00	-	1.00	-
50 - 59	1075(28.26)	14(8.00)	1.91(0.85 - 4.33)	0.118	1.93(0.85 - 4.36)	0.114
60 - 69	792(20.82)	43(24.57)	7.99(3.99 - 15.98)	< 0.001	8.08 (4.04 - 16.18)	< 0.001
70 - 79	400(10.52)	77(44.00)	28.32(14.52 - 55.23)	< 0.001	28.56(14.64 - 55.73)	< 0.001
≥ 80	66(1.74)	31(17.71)	69.09(32.50 - 146.89)	< 0.001	69.32(32.59-147.45)	< 0.001

**Sex**						
Male	1737(45.66)	74(42.29)	1.00	-	1.00	
Female	2067(54.34)	101(57.71)	1.15(0.84 - 1.56)	0.381	1.20(0.87 - 1.66)	0.260

**Education**						
Literate	1781(46.82)	79(45.14)	1.00	-	1.00	-
Illiterate	2023(53.18)	96(54.86)	1.07(0.79 - 1.45)	0.664	1.07(0.77 - 1.47)	0.683

^φ ^Low vision + blind

### Cataract blindness and surgery

Among 17 blind, 8 (47.06%) were blind as a result of cataract in both eyes. The prevalence of bilateral cataract blindness was 0.2% (95% CI. 0.09 - 0.39). Among 143 unilaterally blind, cataract was responsible in 53 (37.5%) subjects representing a prevalence of 1.3% (95%CI. 1.00 - 1.74). Together 61/160 (38.1%) subjects, 1.5% (95% CI. 1.17 - 1.96) of the unilateral and bilateral blind with cataract as the principle cause of blindness in at least one eye could potentially have been helped by cataract surgery.

A total of 151 subjects underwent cataract surgery (Table 5). Pseudophakia was present in 142 (94.0%), males 58 (40.9%) and females 84 (59.1%), while aphakia in 9 (6%). 70 (49.3%) subjects had pseudophakia in one eye, 70 (49.3%) had in both eyes and 2 (1.41%) subjects had aphakia in one eye and pseudophakia in the other. The distribution of 47 individuals who were never operated with cataract blindness (we excluded 14 out of the 53 unilateral blind who had been operated in the fellow eye, none for the bilateral blind group) is presented in table 5. Cataract blindness and surgery was only associated with advancing age. The cataract surgical coverage of the study population was 90.36%. Surgical coverage was higher in the younger age group, females and among those who were illiterate.

**Table 5:** Cataract blindness (VA < 3/60) and cataract surgery prevalence by age, sex and literacy.

				Cataract operated						
**Age**	**No** **examined**	**Never operated** **cataract blind**		**All operated**		**Presumed** **blind**		**Cataract** **blindness burden**		**% Surgical** **coverage**
		
		**No**	**Prevalence**	**No**	**Prevalence**	**No**	**Prevalence**	**No**	**Prevalence**	**No**

40-49	1481	0	0.00	3	0.20	2	0.13	2	0.13	100

50-59	1089	4	0.37	19	1.74 ^i^	14	1.28	18	1.65 ^a^	100

60-69	835	11	1.32	37	4.43 ^ii^	19	2.27	30	3.59 ^b^	94.4

70-79	477	21	4.40	66	13.84 ^iii^	41	8.59	62	13.00 ^c^	85.3

>80	97	11	11.34	26	26.80 ^iv^	19	19.59	30	30.92 ^d^	87.5

**Sex**										

Male	1811	23	1.27	63	3.48	35	1.93	58	3.20	85.3

Female	2168	24	1.10	88	4.06	60	2.77	84	3.87	93.9

**Education**										

Literate	1860	20	1.07	75	4.03	45	2.42	65	3.49	85.71

Illiterate	2119	27	1.27	76	3.59	50	2.36	77	3.63	95.12

All	3979	47	1.18	151	3.79	95	2.39	142	3.57	90.36

^i ^Adjusted odds ratio with 95% C.I. versus age 40 - 49: 8.79 (2.59 - 29.78)

^ii ^Adjusted odds ratio with 95% C.I. versus age 40 - 49: 23.22 (7.14 - 75.58)

^iii ^Adjusted odds ratio with 95% C.I. versus age 40 - 49: 79.47 (24.85 - 254.09)

^iv ^Adjusted odds ratio with 95% C.I. versus age 40 - 49: 182.67 (53.97 - 618.17)

^a ^Adjusted odds ratio with 95% C.I. versus age 40 - 49: 12.54 (2.90 - 54.18)

^b ^Adjusted odds ratio with 95% C.I. versus age 40 - 49: 28.02 (6.68 - 117.58)

^c ^Adjusted odds ratio with 95% C.I. versus age 40 - 49: 111.61 (27.18 - 458.27)

^d ^Adjusted odds ratio with 95% C.I. versus age 40 - 49: 333.54 (78.04 - 1425.51)

### Awareness of cataract and glaucoma

Out of a total of 11,499 subjects that were interviewed complete data was available for 10,303 subjects. In this group 52.32% were males, 61.48% were illiterate and 69.65% belonged to the Newar caste. 55.8% of the total subjects had never undergone an eye examination.

A total of 682 (6.7%) of the subjects were aware of cataract while 244 (2.43%) of glaucoma. Multivariate logistic regression analyses (Table 6) indicated that awareness of cataract did not increase considerably with the increase in age group while for glaucoma the awareness increased significantly except for subjects in the highest age group. For both cataract and glaucoma, awareness was higher among males, literates and in the Brahmin and Chhetri caste groups.

**Table 6:** Association of awareness of cataract and glaucoma with age, sex, literacy and caste (N = 10303)

Variable	Total number	Num aware of Cataract (%)	OR for being aware of Cataract (95% C.I.)	Num aware of Glaucoma (%)	OR for being aware of Glaucoma (95% C.I.)
**Age (yrs.)**^**a**^					
40 - 49	4032	328(8.1)	1.00	118(2.9)	1.00
50 - 59	2707	181(6.7)	1.02 (0.84 - 1.25)	81(3.0)	1.38 (1.02 - 1.85)
60 - 69	1931	106 (5.5)	1.02 (0.80 - 1.30)	53(2.7)	1.65 (1.16 - 2.35)
≥ 70	1633	67 (4.1)	0.97 (0.73 - 1.30)	16(1.0)	0.78 (0.45 - 1.35)
Total	10303	682(6.6)	-	268(2.6)	

**Sex**^**b**^					
Male	4912	398 (8.1)	1.00	157(3.2)	1.00
Female	5391	284 (5.3)	0.63(0.54 - 0.74)	111(2.1)	0.64 (0.50 - 0.81)

**Literacy**^**c**^					
Literate	3968	499 (12.6)	1.00	215(5.4)	1.00
Illiterate	6335	183 (2.9)	0.23(0.18 - 0.28)	53(0.8)	0.15 (0.10 - 0.21)

**Caste**^**d**^					
Brahmin/Chhetri	2396	383 (16.0)	1.00	161(6.7)	1.00
Newar	7177	230 (3.2)	0.22 (0.18 - 0.26)	87(1.2)	0.23 (0.18 - 0.30)
Others	730	69 (9.4)	0.71 (0.54 - 0.94)	20(2.7)	0.55 (0.34 - 0.89)

^a,b,c,d, ^p < 0.001 for cataract and glaucoma each, χ^2 ^test in univariate analysis

Responses to questions on cataract and glaucoma are presented in Table 7 and the questionnaire in Table 8. Of the 682 subjects who were aware of cataract 480(70.38%) also had knowledge of the condition. 423(62.0%) subjects had knowledge that cataract was as an appearance of a 'pearl like dot' white opacity in the eye. Of the 268 subjects who were aware of glaucoma, 122 (45.5%) subjects had knowledge of the condition. 71 (26.49%) subjects had known that glaucoma could cause blindness. Media was the most frequent source of information for both cataract (39.7%) and glaucoma (40.3%).

**Table 7:** Responses among those aware of cataract and glaucoma

What is Cataract/ Glaucoma?	Number (%)		Source of information	Number (%)	
			
	Cataract	Glaucoma		Cataract	Glaucoma
Blurred vision^a^	48 (7.0)	18(6.7)	Family member	86 (12.6)	29 (10.8)

Blindness^b^	6 (0.9)	71(26.5)	Hospital	91 (13.3)	37 (13.8)

Pearl like opacity^c^	423(62.0)	3 (1.1)	Doctor	69 (10.1)	26 (9.7)

Rainbow haloes^d^	3(0.4)	22 (8.2)	Friends	107 (15.7)	32 (11.9)

Raised eye pressure^e^	0(0.0)	8 (2.9)	Media	271 (39.7)	108 (40.3)

Unmatched answers	202 (29.6)	146 (54.5)	Others	58 (8.5)	36 (13.4)

Total	682 (100)	268 (100)	total	682 (100)	268 (100)

^a, b, c, d, e ^considered as knowledge

**Table 8:** Questionnaire on Awareness of Cataract and Glaucoma

Tilganga Institute of Ophthalmology															
Bhaktapur Glaucoma Study															
Questionnaire: Awareness of Cataract and Glaucoma															
Date of Interview															
*Please write the English Date (dd/mm/yr):*															
*Name of Interviewer:*						*Code*					*Ward:*				
House No.	S N	How many years have you been living here? *	Do you have people aged 40 or more in your family?	**(If the answer is yes in the last 2 questions then take the interview of the people aged 40 or more in that family)**	Sex	Caste		Education	Have you had your eyes examined by a doctor?	If yes, where?	Do you know about these eye diseases?		If yes, what do you know about them?		What is your source of information?
									
					M = 1 F = 2	Brahmin/Chhetri = 1 Newar = 2 Tamang = 3 Muslim = 4 Dalit = 5 Others (specify)= 6	Age	Illiterate = 0 < 6 grade = 1 6-10 = 2 > 10 = 3 Others = 4		Tilganga Hospital = 1 Community Eye Centre Bhaktapur = 2 Other Hospital = 3 Private Clinic/Nursing home = 4 Heath camps = 5 Others (specify) = 6	Yes = 1 No = 0		Blurred Vision = 1 Blindness = 2 Pearl like opacity = 3 Rainbow haloes = 4 Raised eye pressure = 5 Unmatched answers = 6		Family members = 1 Hospital = 2 Doctor = 3 Friends/neighbors = 4 Radio/TV/Newspapers = 5 Others(specify) = 6 Don't know = 9
										
		< 1 year = 0 > 1 year = 1	Yes = 1 No = 0	Write the name serially.					Yes = 1 No = 0		Glaucoma	Cataract	Glaucoma	Cataract	

* If the time of stay is less than 1 year/there is no one in the family of 40 or above age - do not interview.

## Discussion

Our data has been presented using WHO criteria for VI in order to compare results with other studies. Data has also been presented based on presenting VA to address the 'real' magnitude of VI in this population.

The overall prevalence of VI was low in Bhaktapur. It was associated with advancing age, female sex prior to best correction and was not associated with literacy. After best correction there was no difference between the sexes. The prevalence of blindness and low vision in Bhaktapur district is lower than reports from studies undertaken in Nepal.

The prevalence of blindness at presentation was 0.73% which is lower than NBS (3.4%), 1995 Lumbini survey (3%) and 2002 Gandaki zone study (1.4%). This is also lower than studies conducted in neighboring countries and the estimate of 3.4% for the South East Asian region [18-22]. However there are several studies in Asia that have also reported a low prevalence of blindness [23-27]. After best correction the prevalence of blindness was 0.43%.

Cataract remains the principle cause of blindness. The prevalence of cataract blindness was 1.5% which is almost similar to the Gandaki zone study (1.9%) where the outcome of the study were from the area best served by the local eye hospital. The major cause of bilateral blindness (53.3%) was cataract which was comparable to other studies in Nepal. Together, cataract (60.8%) and refractive error (12.0%) contributed 72.8% of the total burden of low vision which was curable. There were more women with low vision due to uncorrected refractive error. The 1981 NBS and the 1995 Lumbini survey have also reported that females were more likely to have VI. This finding in our population could suggest that women were not seeking eye care for reasons such as unequal access, social stigma related to wearing spectacles and others. In the future, rehabilitation programs will need to targeted for women among this population.

A cataract surgical coverage (CSC) of 90.36% was highest in comparison to all the other studies of Nepal. Since 1994, TIO has held numerous cataract screening programs in Kathmandu valley particularly focusing on Bhaktapur. These services could have lead to the high CSC. In comparison to populations from other districts of Nepal, people of Bhaktapur have a better access to eye care as there are two community eye centers that are affiliated to tertiary eye hospitals in Kathmandu. A higher CSC among the younger age groups is consistent with reports from other studies in Nepal. This is not unusual considering that younger population is more active and likely to seek earlier treatment. CSC being higher in females was different from previous studies undertaken in Nepal and elsewhere [28,29]. We are unable to explain why there were more females that had undergone surgery.

Among subjects that had undergone cataract surgery, 94% had pseudophakia. This was very high compared to 16.4% seen in Lumbini district [30]. The Fred Hollows Intraocular Lens Laboratory at TIO has been manufacturing intraocular lenses since 1994. The availability and affordability of intraocular lenses could also have lead to a high prevalence in Bhaktapur.

Awareness and knowledge of cataract and glaucoma was very poor. We are alarmed and unable to explain the reason for such a low awareness on cataract despite there being several cataract screening programs held in the past several years in Bhaktapur. Subjects mostly understood cataract as a 'pearl like dot' white appearance in the eye while glaucoma was known to cause blindness. Very few had knowledge of glaucoma as a disease of eye pressure. Previous studies on cataract surgery undertaken in Nepal [31] and south India [32] have reported that males, literates and those affluent were more likely to be aware of cataract surgery. Similarly in our study males, literates and the affluent Brahmin and Chettri [33] classes were more aware of both conditions.

It need not be stressed that patient education programs will have to be incorporated in cataract intervention programs to raise awareness and encourage the people to come forth for an eye examination. Majority of the subjects (55.8%) had never undergone an eye examination.

It is well known that patient education programs have been successful in decreasing the morbidity of diseases [34,35] and have also helped improve compliance in glaucoma patients [36]. A novel approach to screening and patient education has been adopted by TIO to promote awareness, screening and follow up of patients [37].

Bhaktapur is one of the three districts of Kathmandu valley which represents a metropolitan city with a predominantly agrarian rural periphery. It is situated approximately 15 kilometers from Kathmandu the capital city of Nepal. We selected this district because it does not have an eye hospital to serve its population. From findings of this study, it is possible that the other two districts within Kathmandu valley which share similar socioeconomic conditions and geographic terrain could also have a low prevalence of VI. With the availability of eye services in these districts the prevalence of VI could in fact be much lower. However, further population based studies are required to confirm this statement.

The required sample size of 4758 subjects couldn't be fulfilled because we were not able to convince all subjects to undergo an eye examination at the hospital, besides seventy five subjects had also died during the time of the survey. The major strength of our study was the large number of subjects interviewed during the field work and the comprehensive eye examination at the base hospital that resulted in accurate diagnosis.

## Conclusions

The low prevalence of visual impairment and the high cataract surgical coverage suggests that cataract intervention programs have been successful in Bhaktapur. Awareness and knowledge of cataract and glaucoma was very poor in this population. Eye care programs needs to be directed towards preventing visual impairment from refractive errors, screening for incurable chronic eye diseases and promoting health education to raise awareness of cataract and glaucoma in this population

## Competing interests

None of the authors has a financial or proprietary interest in any material or method mentioned.

## Authors' contributions

SST has drafted the manuscript, contributed to the design of the study, analysis and interpretation of data. PP has contributed to the design of the study. RVDB, SK, NM has helped analyze and interpret of data. SNT has helped in the field work. SK, IP, GP, RG, SR and GH.M.B.R have revised the manuscript critically. All authors have read and approved the final manuscript.

## Pre-publication history

The pre-publication history for this paper can be accessed here:

http://www.biomedcentral.com/1471-2415/11/2/prepub
